# Draft Genome Sequence of *Frankia* sp. Strain CN3, an Atypical, Noninfective (Nod^–^) Ineffective (Fix^–^) Isolate from *Coriaria nepalensis*

**DOI:** 10.1128/genomeA.00085-13

**Published:** 2013-03-14

**Authors:** Faten Ghodhbane-Gtari, Nicholas Beauchemin, David Bruce, Patrick Chain, Amy Chen, Karen Walston Davenport, Shweta Deshpande, Chris Detter, Teal Furnholm, Lynne Goodwin, Maher Gtari, Cliff Han, James Han, Marcel Huntemann, Natalia Ivanova, Nikos Kyrpides, Miriam L. Land, Victor Markowitz, Kostas Mavrommatis, Matt Nolan, Imen Nouioui, Ioanna Pagani, Amrita Pati, Sam Pitluck, Catarina L. Santos, Arnab Sen, Saubashya Sur, Ernest Szeto, Fernando Tavares, Hazuki Teshima, Subarna Thakur, Luis Wall, Tanja Woyke, Louis S. Tisa

**Affiliations:** a University of New Hampshire, Durham, New Hampshire, USA; b University of Tunis El Manar, Tunis, Tunisia; c Los Alamos National Laboratory, Los Alamos, New Mexico, USA; d DOE, Joint Genome Institute, Walnut Creek, California, USA; e Oak Ridge National Laboratory, Oak Ridge, Tennessee, USA; f Centro de Investigação em Biodiversidade e Recursos Genéticos, Porto, Portugal; g University of North Bengal, Siliguri, India; h Instituto de Biologia Celular e Molecular, Porto, Portugal; i University of Quilmes, Bernal, Argentina

## Abstract

We report here the genome sequence of *Frankia* sp. strain CN3, which was isolated from *Coriaria nepalensis*. This genome sequence is the first from the fourth lineage of *Frankia*, strains of which are unable to reinfect actinorhizal plants. At 10 Mb, it represents the largest *Frankia* genome sequenced to date.

## GENOME ANNOUNCEMENT

Among the *Actinobacteria*, the genus *Frankia* is well known for its facultative lifestyle as a plant symbiont of dicotyledonous plants and as a free-living soil dweller (1–3). Our understanding of this genus has been greatly enhanced by the sequencing of several *Frankia* genomes representing three different *Frankia* lineages (4–6). Besides these three major lineages of *Frankia* that are known to reinfect their host plants, a fourth *Frankia* lineage (termed atypical *Frankia* isolates) is recognized that represents isolates that are unable to reinfect actinorhizal plants. Although this group forms a clade within *Frankia*, it is phylogenetically distinct from the other three lineages based on several criteria, including the 16S rRNA gene (7), *glnII* (8, 9), and a 16S-23S rRNA intergenic spacer region (10).

*Frankia* sp. strain CN3 was chosen for sequencing and is a member of the fourth *Frankia* lineage and an atyptical *Frankia* strain that is noninfective (Nod^–^) and non-nitrogen fixing (Fix^–^). The bacterium was isolated from root nodules obtained on soil samples collected from Muree, Northern Pakistan, using *Coriaria nepalensis* seedlings (11). *Frankia* sp. strain CN3 is also resistant to elevated levels of toxic heavy metals, including Cu^+2^ (12), and has potential applications in bioremediation. Strain CN3 was sequenced to provide information about the potential ecological roles of the atypical *Frankia* strains and their interaction with actinorhizal plants. Since symbiotic interactions between *Frankia* and the host plant are not well understood at a molecular level, comparative genomics of atypical and infective *Frankia* strains should provide clues on their symbiotic lifestyle.

The draft genome of *Frankia* sp. strain CN3 was generated at the Department of Energy (DOE) Joint Genome Institute (JGI) using a combination of 454-GS-FLX-Titanium (13) and Illumina GAii (14) techniques. A standard 454 Titanium library, which generated 918,729 reads, a paired-end 454 library with an average insert size of 8 kb, which generated 756,574 reads totaling 511.5 Mb of 454 data, and an Illumina GAii shotgun library, which generated 65,924,070 reads totaling 5,010.2 Mb, were created. All techniques for DNA isolation, library construction, and sequencing were performed according to JGI standards and protocols (http://www.jgi.doe.gov). The 454 Titanium standard data and the 454 paired-end data were assembled together with Newbler, version 2.3 (13), and resulted in 379.9 Mb of 454 draft data, which provided an average 38× coverage of the genome. The Illumina sequencing data were assembled with Velvet, version 1.0.13 (15), and the resulting 4,476.3 Mb of Illumina draft data provided an average 447.6× coverage of the genome. For finishing, the gaps and misassemblies were resolved by editing in Consed, by PCR, and by bubble PCR primer walks.

The draft genome of *Frankia* strain CN3 was resolved to 2 scaffolds consisting of 9,978,592 bp with a G+C content of 71.81%, 8,333 candidate protein-encoding genes, 79 tRNA genes, and 3 rRNA regions. Strain CN3 represents the largest *Frankia* genome sequenced to date.

### Nucleotide sequence accession numbers.

The *Frankia* sp. strain CN3 genome sequence has been deposited at DDBJ/EMBL/GenBank under the accession number AGJN00000000. The version described in this paper is the first version, AGJN01000000.
